# A287 LEVERAGING MACHINE LEARNING TO IMPROVE THE DIAGNOSTIC ACCURACY OF ULTRASOUND SCREENING FOR HEPATOCELLULAR CARCINOMA

**DOI:** 10.1093/jcag/gwad061.287

**Published:** 2024-02-14

**Authors:** V Govardhanam, M ZEGHAL, A Cheung

**Affiliations:** University of Ottawa, Ottawa, ON, Canada; University of Ottawa, Ottawa, ON, Canada; University of Ottawa, Ottawa, ON, Canada

## Abstract

**Background:**

Ultrasound screening stands out as the gold standard for hepatocellular carcinoma (HCC) detection, attributed to its broad accessibility, patient-friendly, and cost-efficient nature. Nevertheless, the five-year survival rate for HCC currently rests at 32.7%, with suboptimal screening being a key contributor to this. The recent years have witnessed the rise of machine learning models, powered by artificial neural networks. Among these, convolutional neural networks (CNNs) have taken the lead in revolutionizing medical image analysis, offering unprecedented success in predictive tasks and giving us hope for a brighter future in the fight against HCC

**Aims:**

To train and test a machine learning algorithm using pre-trained CNNs to improve early detection of HCC through ultrasound screening.

**Methods:**

In this retrospective study, 1835 charts of patients with chronic liver disease were reviewed: 346 with histologically confirmed HCC and 1457 with ultrasounds without HCC. A diagnosis of HCC was confirmed pathologically on biopsy or surgical resection, and/or radiographically with a Liver Imaging Reporting and Data System (LI-RADS) score of five on CT and/or MRI. Patients with benign lesions were required to have at least two ultrasounds three years apart that confirmed benign characteristics. Cases were excluded if they had a prior history of treated HCC, post-transplant HCC, or HCC with Barcelona Clinic Liver Cancer (BCLC) Stage B and above. All ultrasound images were reviewed by experienced radiologists, and segmented as liver lesions (HCC versus benign) and surrounding liver.

**Results:**

A total of 149 patients have been included to date, comprising 72 with benign lesions, 73 with HCC, and four with both benign and malignant lesions. 224 lesions have been segmented, consisting of 87 HCC and 137 benign lesions. Candidate networks are under development and evaluation for the classification of liver lesions. Imaging pre-processing was performed such that the liver region of interest (ROI) and the lesion ROI were standardized, with 2-channel greyscale images on two separate channels. The algorithm was constructed using a per-lesion analysis. Initial testing has achieved an area under the curve (AUC) of 73.3%, 95% CI [68.6, 77.0] with 2 repetitions of 10-fold cross-validation.

**Conclusions:**

Enhancing ultrasound screening for HCC is imperative for improving patient care. Analysis of remaining cases is ongoing. Future studies will be essential, including prospective evaluation and external validation.

**Funding Agencies:**

None

